# *Carnobacterium divergens* Bacteremia in Woman

**DOI:** 10.3201/eid2106.141799

**Published:** 2015-06

**Authors:** Mounira Smati, Christia Palacios, Yves Cohen, Frédéric Méchaï, Jacques Tankovic, Anne Le Flèche-Mateos, Bertrand Picard, Frédéric Gonzalez

**Affiliations:** Avicenne University Hospital, Bobigny, France (M. Smati, C. Palacios, Y. Cohen, F. Méchaï, B. Picard, F. Gonzalez);; Saint-Antoine University Hospital, Paris, France (J. Tankovic);; Pasteur Institute, Paris (A. Le Flèche-Mateos)

**Keywords:** Carnobacterium divergens, bacteremia, bacterial translocation, probiotics, enteral nutrition, bacteria

**To the Editor:** *Carnobacterium* spp. are ubiquitous lactic acid bacteria isolated from cold and temperate environments (*1*). They are present in food including fish, meat, and dairy products. Only *C. divergens* and *C. maltaromaticum* (formerly *C. piscicola*) are found in dairy products (*2*). Carnobacteria are well known for their ability to produce bacteriocins that inhibit *Listeria monocytogenes* (*1*). Because *Carnobacterium* and *Listeria* bacteria are psychrotrophic and share the same ecologic niche, many studies have highlighted the potential use of carnobacteria as a biopreservative (*1*). These bacteria were previously believed to be nonpathogenic for humans. We report a case of *C. divergens* bacteremia in a woman.

In January 2013, a 57-year-old woman with a history of diabetes mellitus, severe undernutrition, and chronic alcoholism was admitted to the intensive care unit of the Avicenne Hospital, Bobigny, France, for diabetic ketoacidosis with altered level of consciousness. Physical examination revealed a low body temperature (30.1°C) and epigastric tenderness. At admission, a computed tomographic scan of the abdomen showed pneumoperitoneum with low-abundance ascites. Antimicrobial therapy with piperacillin/tazobactam and amikacin was empirically started. Exploratory laparotomy findings were within normal limits. 

Three days after admission, acute necrotizing esophagitis (“black esophagus”) with multiple gastroduodenal ulcerations was diagnosed by gastrointestinal endoscopy. By then, septic shock had developed. Antimicrobial drug therapy was empirically changed to imipenem/cilastatin and amikacin. A total esophagectomy with gastrostomy and esophagostomy was performed. No etiology for black esophagus could be established. Parenteral nutrition was begun 24 hours after surgery and relieved with enteral nutrition 72 hours after surgery. On hospitalization day 13, after having clinically improved, the patient consecutively experienced 2 episodes of hypoxemic cardiac arrest and resuscitation. Fever began 2.5 hours later and septic shock again developed. Exploratory laparotomy findings ruled out ischemic colitis.

Four sets of blood cultures collected on 3 days over a period of 5 days showed bacterial growth after 2 days of incubation in the BACTEC 9240 System (Becton Dickinson, Franklin Lakes, NJ, USA). Gram-positive *Listeria*-like rods were seen. Within 24 hours, the isolate grew on trypticase soy agar with 5% horse blood and chocolate PolyViteX agar (bioMérieux, Marcy l’Étoile, France). The colonies were gray, 1–2 mm in diameter, and nonhemolytic. The strain was facultative anaerobic. The catalase reaction was negative, and the esculin hydrolysis reaction was quickly positive. Results of testing with the API Coryne and API Listeria systems (bioMérieux) were unclear. The isolate seemed to be susceptible to penicillins, carbapenems, macrolides, and gentamicin and resistant to cephalosporins. MICs were as follows: penicillin 0.19 mg/L, amoxicillin 0.125 mg/L, amoxicillin/clavulanic acid 0.094 mg/L, cefotaxime >32 mg/L, ofloxacin 1 mg/L, ciprofloxacin 0.38 mg/L, imipenem 0.064 mg/L, vancomycin 2 mg/L, teicoplanin 1 mg/L, linezolid 0.50 mg/L, amikacin 16 mg/L, and rifampin 0.006 mg/L.

Because blood cultures were positive for gram-positive rods susceptible to amoxicillin, our initial diagnosis was listeriosis. Empirically prescribed antimicrobial therapy (ceftazidime, colistin, amikacin, and metronidazole) was given for 96 hours and then replaced by gentamicin for 48 hours and amoxicillin for 3 weeks; clinical results were favorable.

The isolate strain was analyzed by the Division of Bacterial Identification (Pasteur Institute, Paris, France). The 16S rRNA gene was completely sequenced. A phylogenetic tree was generated by using the neighbor-joining algorithm (*3*). The isolate was found be *C. divergens*. Microbiological cultures and 16S rRNA testing results for another sample of enteral nutrition solution and a surgical specimen of the necrotic esophagus were negative.

Three reports of isolation of *Carnobacterium* sp. from humans have been published. The first report described isolation of *Carnobacterium* sp. from 1 set of blood cultures from a man who had prepared fish before onset of fever (*4*). The imputability of this diagnosis could not be clearly established because only 1 set of blood cultures had positive results. The second report described isolation of *C. piscicola* from pus after traumatic amputation of a hand by an industrial water sawmill (*5*). The third report described isolation from a child’s hand with multibacterial synergistic gangrene (*6*).

For the case described here, the presence of *C. divergens* in blood cultures cannot be considered contamination because it was isolated from 4 sets of blood cultures collected over 5 days. We hypothesize that bacterial translocation was caused by low mesenteric flow after 2 episodes of cardiac arrest. Because the patient was receiving exclusively enteral nutrition, we presume that the origin of the infection was bacterial contamination of the solution or colonization of the feeding tube. Carnobacteria and lactobacilli (which are used as probiotic bacteria or fermented food products) are similar in that each is found in food, can be used as a biopreservative, and is considered nonpathogenic. The pathogenic relevance of lactobacilli is uncommon, but some clinical infections have been reported, including septicemia and meningitis (*7*). Because *C. divergens* seems to be able to cause life-threatening infection in immunocompromised patients, its safe use in such patients and in the food industry should be monitored.
